# ^18^F-FDG-PET/CT in radiation therapy-induced parotid gland inflammation

**DOI:** 10.1186/s41824-020-00091-x

**Published:** 2020-12-01

**Authors:** Alaa Mouminah, Austin J. Borja, Emily C. Hancin, Yu Cheng Chang, Thomas J. Werner, Samuel Swisher-McClure, Jonathan Korostoff, Abass Alavi, Mona-Elisabeth Revheim

**Affiliations:** 1 Department of Radiology, University of Pennsylvania, Philadelphia, PA USA; 2 The University of Pennsylvania School of Dental Medicine, Philadelphia, PA USA; 3 Perelman School of Medicine at the University of Pennsylvania, Philadelphia, PA USA; 4 Lewis Katz School of Medicine at Temple University, Philadelphia, PA USA; 5 Department of Radiation Oncology, University of Pennsylvania, Philadelphia, PA USA; 6 Division of Radiology and Nuclear Medicine, Oslo University Hospital, Oslo, Norway; 7 Institute of Clinical Medicine, Faculty of Medicine, University of Oslo, Oslo, Norway

**Keywords:** PET/CT, 18F-FDG, Radiation therapy, Parotid gland, Parotid gland inflammation, Head and neck cancer

## Abstract

**Background:**

^18^F-fluorodeoxyglucose-positron emission tomography/computed tomography (FDG-PET/CT) is used in the clinical management of oncologic and inflammatory pathologies. It may have utility in detecting radiotherapy (RT)-induced damage of oral tissues. Thus, the aim of the present study was to use FDG-PET/CT to evaluate parotid gland inflammation following RT in patients with head and neck cancer (HNC).

**Methods:**

This retrospective study included patients with HNC treated with photon, proton, or combined photon/proton RT, in addition to chemotherapy. All patients received FDG-PET/CT imaging pre-treatment and 3 months post-treatment. The average mean standardized uptake value (Avg SUVmean) and the average maximum standardized uptake value (Avg SUVmax) of the left and right parotid glands were determined by global assessment of FDG activity using OsiriX MD software. A two-tailed paired *t* test was used to compare Avg SUVmean and Avg SUVmax pre- and post-RT.

**Results:**

Forty-seven HNC patients were included in the study. Parotid gland Avg SUVmean was significantly higher at 3 months post-treatment than pre-treatment (*p* < 0.05) in patients treated with photon RT, but no significant differences were found between pre- and post-treatment Avg SUVmean in patients treated with proton RT or combined photon/proton RT.

**Conclusion:**

Our results suggest that photon RT may cause radiation-induced inflammation of the parotid gland, and that proton RT, which distributes less off-target radiation, is a safer treatment alternative.

## Background

Head and neck cancers (HNC) represent approximately 4% of all cancers in the USA (Head and Neck Cancer - Statistics, 2012). This group of malignancies affects a variety of anatomic structures, including the oral cavity, oropharynx, nasopharynx, hypopharynx, larynx, paranasal sinuses, and salivary glands (Alterio et al., 2019). Along with surgical resection and/or chemotherapy, HNC may be treated with radiation therapy (RT) as either definitive or adjuvant treatment (Alterio et al., 2019).

The majority of radiation treatment modalities for HNC consist of external beam photon therapy, which has been associated with many systemic sequelae including pneumonitis and vasculitis (Giuranno et al., 2019; Chrapko et al., 2016). Oral complications of RT have proven to be very common in cancer patients, especially those with HNC (Chen, 2019). Critical anatomical structures in close proximity to the irradiated area are often affected during treatment resulting in complications including fibrosis, taste changes, dental caries, periodontal disease, and oral mucositis (Alterio et al., 2019; Shunmuga Sundaram et al., 2019). Because these alterations can have adverse effects on the lifestyle and health outcomes of HNC patients, it is critical to establish an understanding of the off-target effects of RT on the oral region, which contains tissues that are particularly susceptible to radiation-induced damage.

The parotid gland is the largest of the salivary glands and produces 60 to 65% of the total saliva in the oral cavity. It is wrapped around the ramus of the mandible in humans and may be an unintentional target in RT for HNC. Inflammation of the parotid gland has been demonstrated to induce xerostomia resulting in dryness of the oral cavity (Dirix & Nuyts, 2010; Mortazavi et al., 2014). Of note, radiation-induced xerostomia is the most frequently reported complications of RT for HNC and significantly affects patients’ quality of life (Langendijk et al., 2008).

Traditional imaging modalities such as computed tomography (CT) and magnetic resonance imaging (MRI) have been used for staging and monitoring structural changes. In contrast, positron emission tomography (PET) is frequently used to visualize physiological and molecular changes (Buchbender et al., 2012). ^18^F-fluorodeoxyglucose (FDG) is the most commonly used tracer for PET scanning. It is a radiolabeled glucose analog, taken up by cells that rapidly consume and metabolize glucose, such as cancer and inflammatory cells (Love et al., 2005; Rege et al., 1993; Fletcher et al., 2008). The fused FDG-PET/CT allows for detection and quantification of glucose metabolism on the molecular level leading to a more accurate detection of malignancies and inflammatory changes in the head and neck (Beichel et al., 2019; Castaldi et al., 2013). Much of the existing literature on the clinical application of FDG has focused on the radiotracer’s utility in diagnosing malignancies due to the typically increased glucose metabolism of cancer cells, but FDG has also been used for decades to detect inflammatory processes (Borja et al., 2020a). Therefore, we predict that FDG-PET/CT will show potential in the evaluation of radiation-induced inflammation in the parotid gland that predisposes patients to xerostomia. The aim of the present study is to demonstrate the feasibility of FDG-PET/CT in the detection and quantification of the inflammatory effect of RT on the parotid gland in HNC patients.

## Methods

### Patient population

Between February 9, 2010 and November 27, 2018, 64 patients with HNC were treated with photon, proton, or combined photon/proton RT, in addition to chemotherapy with either cisplatin or cetuximab at the University of Pennsylvania. All patients were imaged pre- and 3 months post-treatment with FDG-PET/CT. Of the 64 patients, 17 were not included in the study due to technical issues associated with their FDG-PET/CT scans, inferior imaging quality in the head and neck region and/or mismatch between PET and CT images. The collected clinical data included age, sex, and primary tumor location. The primary tumor locations were tongue, larynx, oropharynx, nasopharynx, and hypopharynx. PET/CT scans used for the study were free from background noise, scatter, and metal artifacts. The study was approved by the Institutional Review Board. It was conducted in compliance with the Health Insurance Portability and Accountability Act (HIPAA).

### FDG-PET/CT image acquisition

All subjects were injected intravenously with 5.0 MBq/kg FDG. After approximately 60 min, FDG-PET/CT images were obtained using the same standardized protocol. Imaging was performed on hybrid PET/CT scanners with comparable spatial resolution (Siemens Biograph 64 mCT (Siemens Healthineers AG, Chicago, IL, USA) and Philips Gemini TF 16 (Philips Medical Systems, Andover, MA, USA)). The images were acquired in accordance with international guidelines (Delbeke et al., 2006; Boellaard et al., 2015) and the institutional PET/CT protocol, including quality control, calibration, and harmonization of PET/CT scanners and validation of standardized uptake value (SUV) measurements. Patients fasted for at least 6 h prior to scanning, and serum glucose levels were immediately measured prior to FDG injection. Three acquisition protocols were used: one for body mass index (BMI) under 30, another for BMI between 30 and 35, and the third BMI over 35; the CT settings were 50, 100, and 150 mAs, respectively, and all at 120 kVp. For the PET acquisitions, the time per bed was 1.5, 2, and 3 min, respectively. Low-dose CT imaging was performed for anatomic localization and attenuation correction. PET images were corrected for scattering, attenuation, scanner dead time, and random coincidences.

### FDG-PET/CT image analysis

**Fig. 1:**
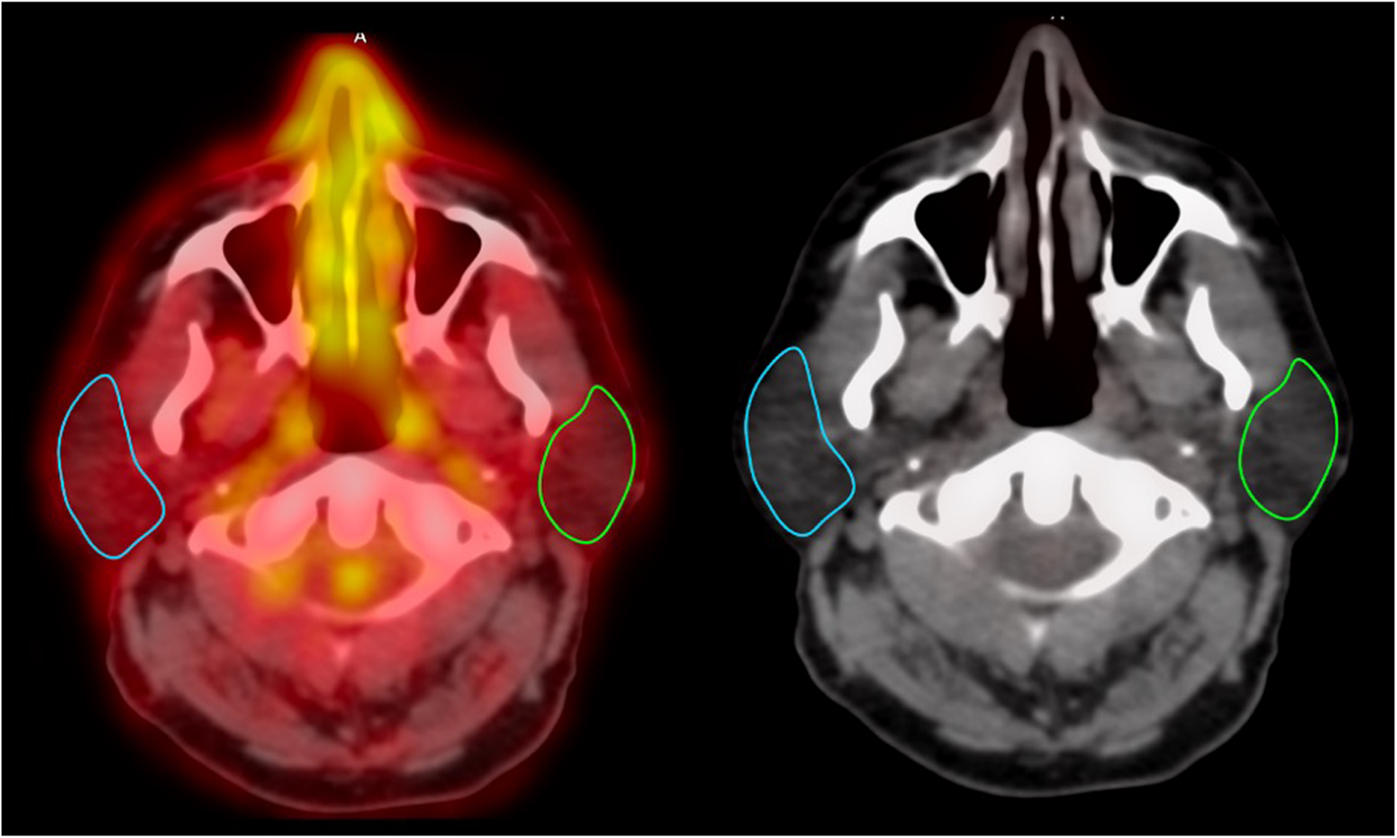
^18^F-fluorodeoxyglucose positron emission tomography/computed tomography (FDG-PET/CT) images of the parotid gland. Left: Fused FDG PET/CT, right: CT. The delineation of the region of interest (ROI) is highlighted for the right and left parotid glands

FDG-PET/CT scans were analyzed using the OsiriX MD software v.10.0.2 (DICOM viewer and image-analysis program, Pixmeo SARL; Bernex, Switzerland). Sequential axial PET/CT slices were used to draw regions of interest (ROI) manually around the right and left parotid glands using a closed polygon (Fig. 1). The reader was blinded to the paired PET scans (pre- and post-treatment scans). Parotid gland ROIs were drawn beginning superiorly at the level of condyle down to the angle of the mandible inferiorly. The skin and external ear were defined as the lateral borders, the styloid process of the temporal bone was the medial border, and the mastoid process of the temporal bone was the posterior border.

The SUVmean was calculated as the average value of all voxels in the ROI. To determine the global activity of the parotid gland, the SUVmean as well as area (mm^2^) of each ROI at each transverse slice was measured and recorded. The SUVmean was multiplied by the area, and the products were summed, the result of which was divided by the sum of the total area of the ROIs (Sum (SUVmean×Area))/(Sum Area). This resulted in the average SUVmean (Avg SUVmean) representative of a global inflammation burden of the parotid gland. The SUVmax was defined as the hottest voxel within the ROI. The average SUVmax (Avg SUVmax) represented the average value from all trans-axial slices, which included both right and left parotid glands. Statistical comparison was completed by using the Avg SUVmean and Avg SUVmax of all slices.

### Statistical analysis

For each subject, pre- and post-treatment Avg SUVmean and Avg SUVmax were calculated. A two-tailed paired *t* test in the STATA software (Stata/IC Version 10.1, StataCorp, College Station, TX) was used to compare the Avg SUVmean and Avg SUVmax in the pre- and post-treatment scans. The level of significance was defined as a *p* value of less than 0.05. The average mean increases in SUVmean and SUVmax were calculated by subtracting pre-treatment from post-treatment Avg SUVmean and Avg SUVmax values for each patient.

## Results

The data collected from a total of 47 HNC patients (25 males, 22 females), mean age 59.7 years (range 42-78) with pre and post-treatment FDG-PET/CT were included. Thirty-three patients were in the photon RT group, while seven patients were in each of the proton RT and combined photon/proton RT groups. Primary tumor location, age, gender, and race are summarized in Table 1.

**Table 1:** Primary tumor location, age, gender, and race

Primary tumor location	Number of patients		Average age (years)	Race		
	Males	Females		White	African American	Other
Tongue	14	3	61.97	15	2	0
Larynx	3	2	54.66	4	0	1
Oropharynx	3	14	58.70	13	3	1
Nasopharynx	3	2	58.25	3	1	1
Hypopharynx	2	1	62.73	2	0	1

**Fig. 2:**
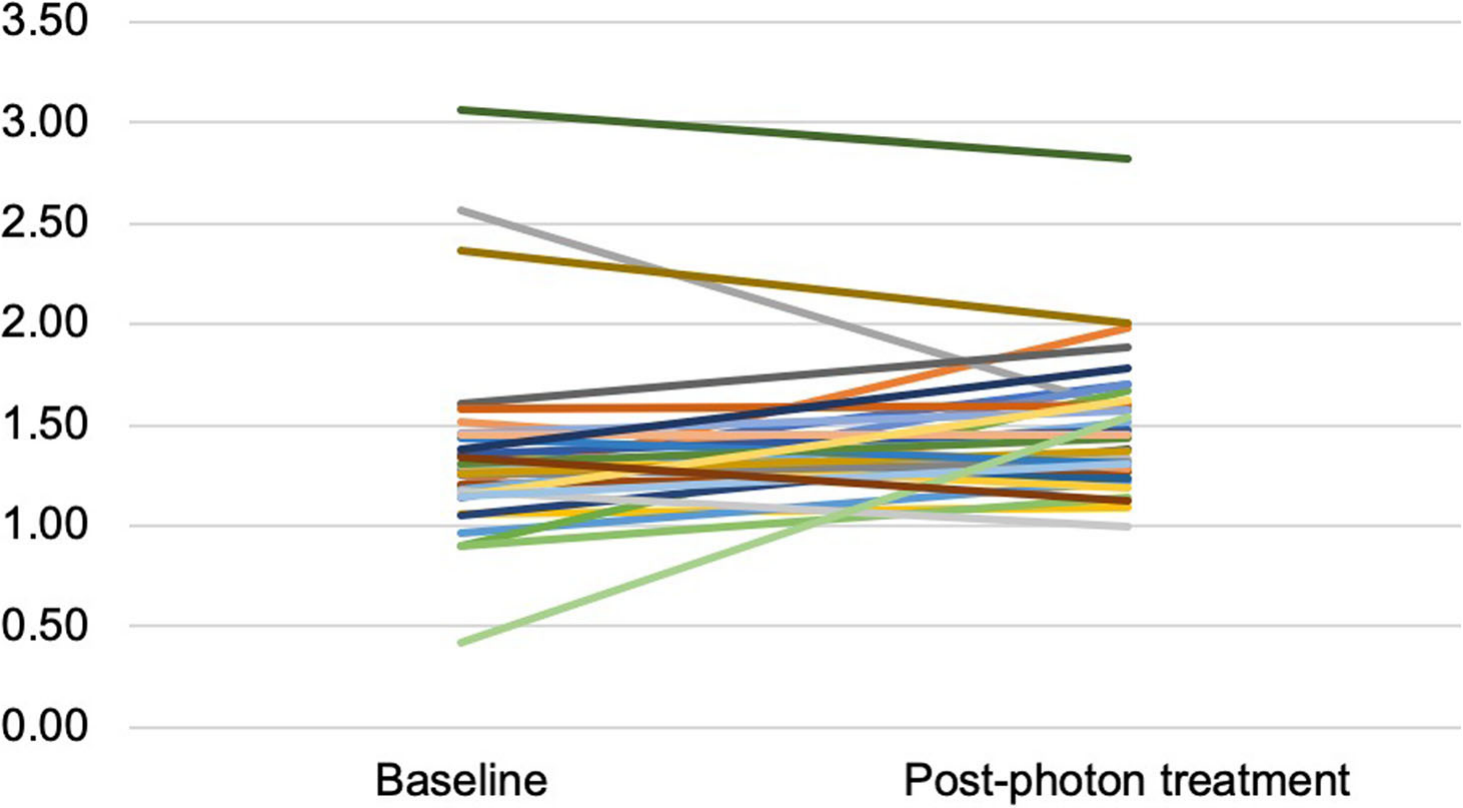
Changes in average standardized uptake value mean (Avg SUVmean) of the parotid gland before and 3 months after treatment in patients treated with photon RT

Statistical data are summarized in Tables 2 and 3. The parotid gland Avg SUVmean in patients treated with photon RT was significantly higher in post-treatment scans (1.50, *p* < 0.05) relative to those done pre-treatment (1.38, *p* < 0.05) (Fig. 2). The Avg SUVmax was higher in post-treatment scans (2.06) compared to those done pre-treatment (1.96), but the difference was not statistically significant.

**Table 2:** Parotid gland average mean standardized uptake values (Avg SUVmean) in pre- and post- treatment scans of head-and-neck cancer patients

Avg SUVmean (g/mL)	Pre-treatment	Post-treatment	***P*** value
**Photon therapy**	1.38	1.50	0.03
**Proton therapy**	1.25	1.32	0.31
**Combined therapy**	1.51	1.46	0.40

**Table 3:** Parotid gland average maximum standardized uptake values (Avg SUVmean) in pre- and post- treatment scans of head-and-neck cancer patients

Avg SUVmax (g/mL)	Pre-treatment	Post-treatment	***P*** value
**Photon therapy**	1.96	2.06	0.18
**Proton therapy**	1.72	1.73	0.50
**Combined therapy**	1.90	2.12	0.21

**Fig. 3:**
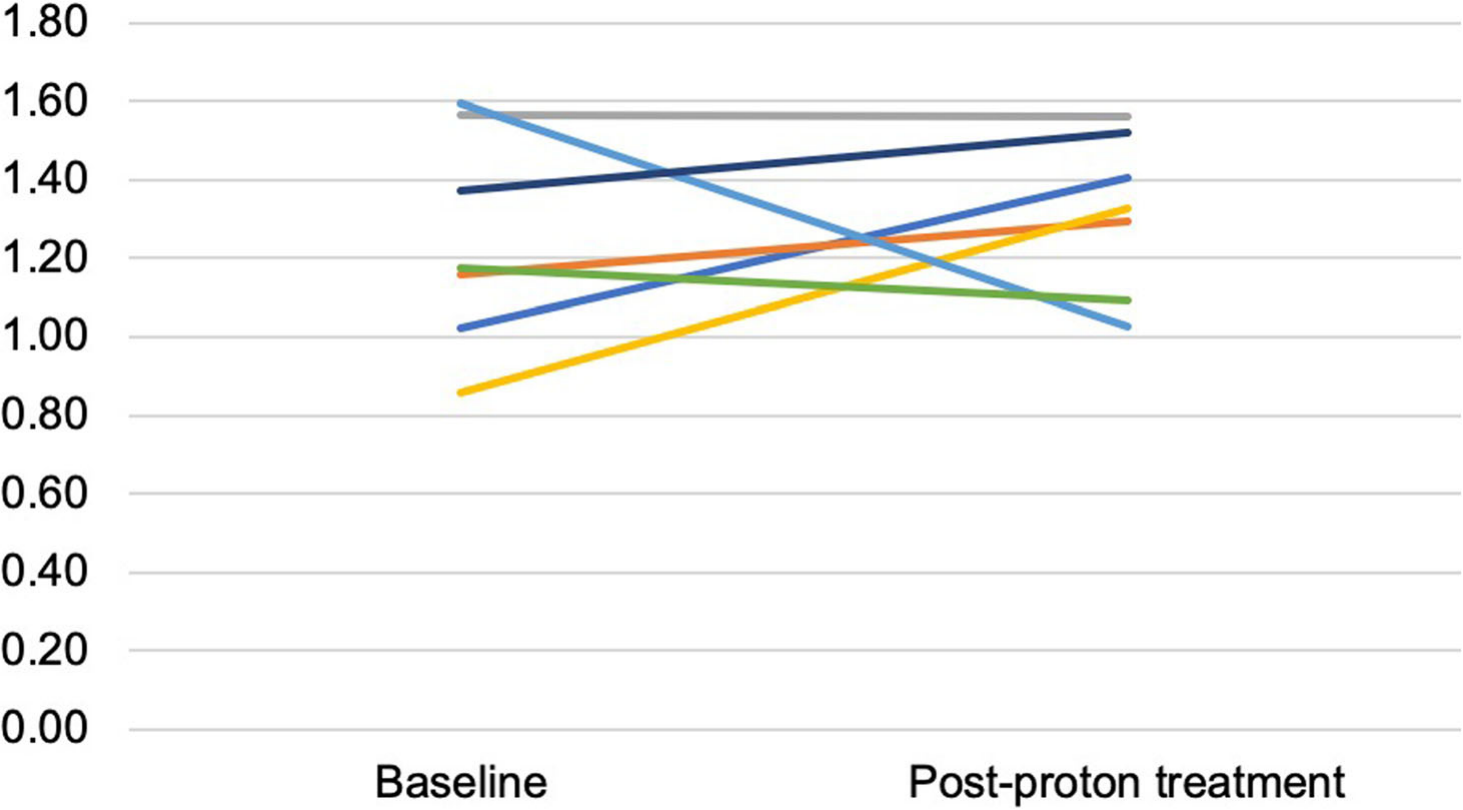
Changes in average standardized uptake value mean (Avg SUVmean) of the parotid gland before and 3 months after treatment in patients treated with proton RT

In patients treated with proton RT, the parotid gland Avg SUVmean was not significantly different in post-treatment scans (1.32, *p* > *0.05*) when compared to pre-treatment scans (1.25, *p* > 0.05) (Fig. 3). Evaluation of pre- and post-treatment scans for Avg SUVmax yielded a similar finding (post-treatment [1.73, *p* > 0.05] and pre-treatment [1.72, *p* > 0.05]).

**Fig. 4:**
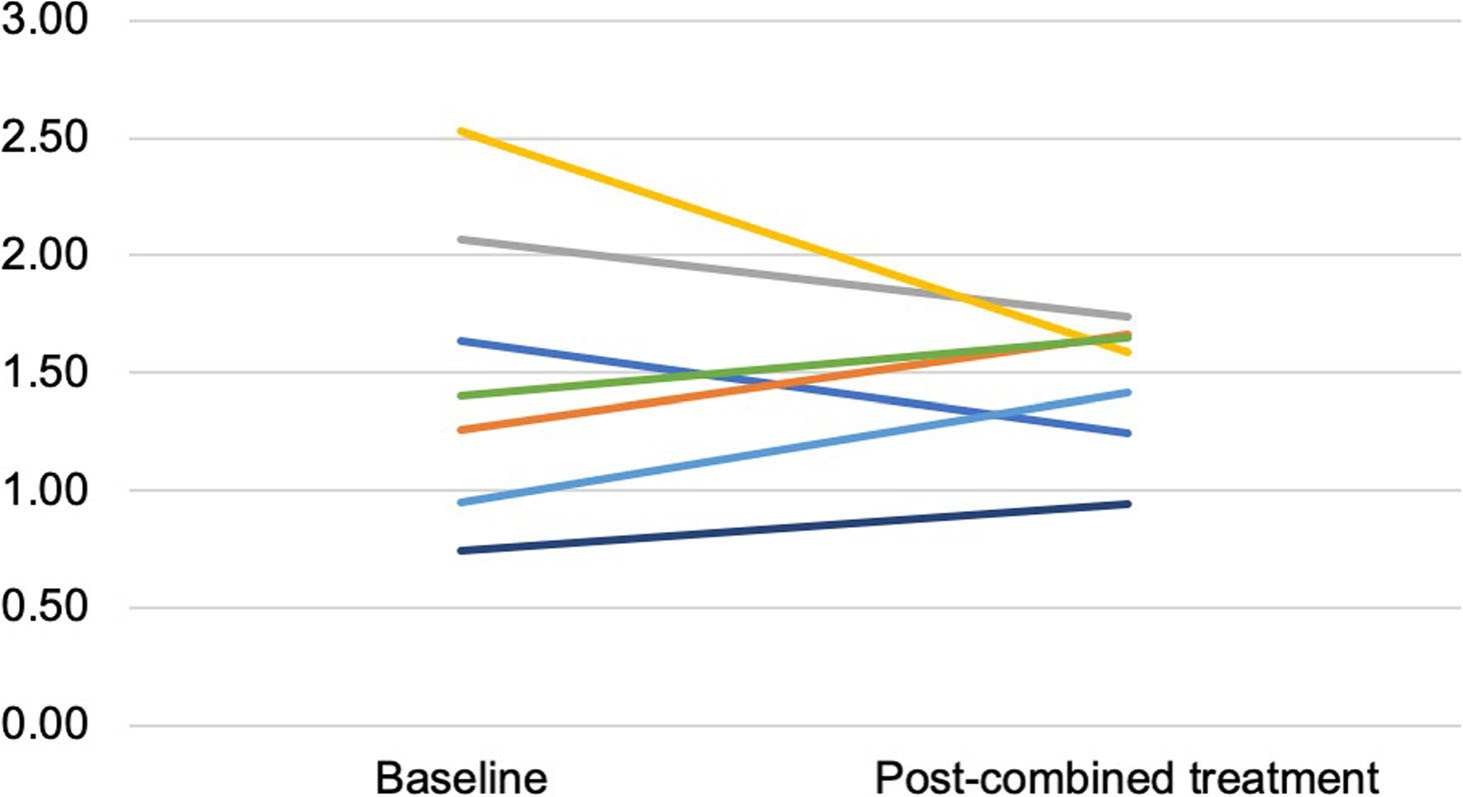
Changes in average standardized uptake value mean (Avg SUVmean) of the parotid gland before and 3 months after treatment in patients treated with combined photon/proton RT

Analysis of the parotid gland Avg SUVmean in patients treated with combined photon/proton RT was not significantly different in post-treatment scans (1.32, *p* > 0.05) when compared to pre-treatment scans (1.25, *p* > 0.05) (Fig. 4). Similar findings were presented for Avg SUVmax (post-treatment [2.12, *p* > 0.05] and pre-treatment [1.90, *p* > 0.05]).

## Discussion

Our study demonstrates a significant increase in FDG uptake in the parotid glands of HNC patients following photon RT and chemotherapy treatments. Investigating the impact of these treatments on the parotid gland is critical, not only because of its role in saliva production but also due to the fact that cranial nerve VII (facial nerve) lies in close proximity to and innervates the gland. This nerve also innervates numerous muscles of facial expression, as well as the stylohyoid and posterior belly of the digastric muscles, which play a critical role in swallowing (Dulak & Naqvi, 2019). The glossopharyngeal nerve provides parasympathetic innervation to the parotid gland, as well as sensory innervation to the posterior one-third of the tongue and pharynx. Photon RT and/or chemotherapy-induced damage of the parotid gland can impact glossopharyngeal nerve function and indirectly lead to deleterious effects on adjacent structures in the head and neck region (García Santos et al., 2018). Thus, determining the FDG uptake in the parotid gland is of clinical importance in investigating potential previously underappreciated side effects of radiotherapy in HNC cancer patients.

In our study, we found that Avg SUVmean was significantly higher in the parotid gland following photon RT, but not in patients who underwent proton RT or combined proton/photon RT. Although Avg SUVmean was significantly increased in patients receiving photon RT, SUVmax values were not significantly different in pre-treatment versus post-treatment scans of patients receiving any form of RT. SUVmax is the maximum voxel value of SUV in the target structure/ROI. SUVmax is simple and observer independent; hence, SUVmax is the most commonly used parameter in clinical practice. However, SUVmax does not represent an entire structure’s metabolic burden because the value is from only one voxel. Furthermore, SUVmax is sensitive to image noise, and is therefore impacted by various patient characteristics and imaging parameters. On the other hand, Avg SUVmean accounts for all uptake within the ROIs and is more reflective of the total pathological changes in glucose metabolism, which suggests that Avg SUVmean is a more accurate value to use in this data collection. Since we suspect that radiation-induced parotid injury is a diffuse pathology that has the potential to elicit an inflammatory response across the entire gland, we used the Avg SUVmean, as it is likely to be a more accurate indicator of the extent of the global inflammation (Høilund-Carlsen et al., 2019; Borja et al., 2020b).

There are several artifacts encountered in PET/CT imaging including attenuation correction artifacts commonly associated with the use of CT. Attenuation correction algorithms work well for most applications in the majority of patients. However, these algorithms tend to overcorrect objects that have higher density but are not true bone pixels. Dental implants or fillings can cause such an attenuation correction artifact and can confound image interpretation and affect the quantification in the head and neck region. In the present study, of the 64 patients, 17 were not included in the study due to technical issues including the presence of artifacts related to metallic based restorations, orthodontic appliances, and other dental procedures, which are the main cause of beam hardening. This confirms the lack of beam hardening artifact effect on our measurements.

We assert that the increased FDG uptake observed in this study was a result of RT-induced inflammation in the parotid gland. Cellular uptake of FDG is a marker for inflammation, and these results confirm its utility in identifying parotid gland pathology following RT in HNC patients. In classic parotitis, this inflammation is most often the result of a localized infection or cellular damage, though the irritation can be caused by a myriad of factors, including pathogenic microbes derived from the oral cavity, metabolic imbalances, and autoimmune disorders (Patel et al., 2017). Initiation of inflammatory processes in the gland can lead to a decrease in salivary production, causing dehydration of the gland as well as a distortion of the parotid duct and metaplasia of the ductal epithelium (Chitre & Premchandra, 1997). Uptake of FDG may begin to increase subsequent to the preliminary irritation and continue to increase as the inflammatory response progresses (Chitre & Premchandra, 1997). Since RT has been shown to increase systemic inflammation, patients experience a significant risk in the perturbation of the parotid gland, since it is particularly susceptible to irritation (Brook, 1992; Schaue et al., 2015).

It is critical to acknowledge the limitations of our study. This was a retrospective analysis with a relatively small sample size of patients. Thus, future evaluation of the use of FDG-PET/CT as a surrogate measurement of inflammatory activity in the parotid gland after RT treatment should be directed toward prospective studies using large numbers of patients. Information regarding full tumor stage, type of radiation field, the exact dosage of radiotherapy administered to patients, and oral complications were not available for the current study, which limited the description of our patient cohort. A survey reported that 64% of at least 3 years survivors after RT suffered from moderate to severe xerostomia (Wijers et al., 2002). Thus, future studies must include detailed information regarding the occurrence of xerostomia in order to determine whether increased parotid-uptake of FDG can be used to predict the onset of this condition. The partial volume effect, which accounts for signal overlap from neighboring anatomical structures and potential movement of the patients during scan acquisition, may have altered the data used in these analyses. Therefore, the regions used as borders in determining the extent of the ROIs may have been ambiguous, depending on the quality of the scan. The influence of partial volume effect is due to the limited resolution of the technology used in obtaining these scans (Cysouw et al., 2016; Soret et al., 2007). This could account for the single outlier observed in the data, which might have introduced further uncertainty into the results (Fig. 2). In addition, because the patients who participated in the study received both chemotherapy and photon RT, it is not possible to differentiate between the inflammatory effects of each treatment individually. Finally, there were only two-time points assessed in this study, pre-treatment and 3 months post-treatment, which prevented the evaluation of FDG uptake throughout the entire post-treatment period.

The present study suggests that an increase beyond normal physiological glucose uptake in the parotid gland occurs as a manifestation of RT-induced inflammation. Given that inflammation is followed by cell damage and fibrosis of some of the glandular tissue (Wynn & Ramalingam, 2012), we predict that additional follow-up scans will demonstrate a decrease in the parotid gland uptake due to lack of normal gland activity and function. It would be helpful to direct future studies toward more longitudinal assessments of FDG uptake in the parotid gland to better track changes in signaling over time to determine the time frame of cell damage and fibrosis manifesting as a decline in parotid gland function compared to pre-treatment.

Protocols utilizing photon beams are currently the most common form of RT for HNC, while less than 1% of patients are treated with proton therapy (Mohan & Grosshans, 2017). When comparing proton to photon therapy, proton therapy reveals an added advantage of lower dose and smaller number of beams (Levin et al., 2005). In the present study, no significant differences were found between pre- and post-treatment parotid FDG uptake in patients treated with proton RT. This observation might be indicative of there being less radiation delivered to normal tissues in close proximity to actual tumors, thereby minimizing collateral toxicity and limiting the extent of side effects traditionally associated with photon-based RT (Mohan & Grosshans, 2017; Lin, 2012). Considering the small patient population receiving only proton therapy, the result must be interpreted with caution and should be confirmed in further studies.

The present study demonstrated significantly higher FDG uptake in the parotid glands of patients undergoing photon-based RT for treatment of HNC. This increase in glucose metabolism may be indicative of radiation-induced inflammation, which subsequently can progress result in decreased functionality of the parotid gland. Future studies should include a larger sample to allow comparison of the effect of photon RT for treatment of HNC to other modalities of RT in order to assess the differential impact on parotid gland function. Confirmation of the correlation between FDG uptake and saliva production might enable clinicians to choose alternative RT regimens and/or intervene at an earlier stage and prevent the sequela of xerostomia.

## Conclusion

FDG-PET/CT has the potential to be used to measure the metabolic activity associated with RT-induced inflammation and predict parotid gland dysfunction.

## Data Availability

All data generated or analyzed during this study are included in this article [and its supplementary information files].

## Abbreviations

FDG-PET/CT: ^18^F-fluorodeoxyglucose-positron emission tomography/computed tomography
RT: Radiotherapy
HNC: Head and neck cancer
CT: Computed tomography
MRI: Magnetic resonance imaging
PET: Positron emission tomography
FDG: ^18^F-fluorodeoxyglucose
HIPAA: Health Insurance Portability and Accountability Act
SUV: Standardized uptake value
Avg SUVmean: Average mean standardized uptake value
Avg SUVmax: Average maximum standardized uptake value
ROI: Region of interest
BMI: Body mass index
